# The Time Course of Catecholamine Dose Reduction in Septic Shock as a Predictor of Bacterial Susceptibility to Empiric Antimicrobial Therapy: A Retrospective Observational Study

**DOI:** 10.3390/jcm13216618

**Published:** 2024-11-04

**Authors:** Tsukasa Kuwana, Kosaku Kinoshita, Yurina Yamaya, Ken Takahashi, Junko Yamaguchi, Atsushi Sakurai, Toru Imai

**Affiliations:** 1 Division of Emergency and Critical Care Medicine, Department of Acute Medicine, Nihon University School of Medicine, Tokyo 173-8610, Japan; kuwana.tsukasa@nihon-u.ac.jp (T.K.); yamaya.yurina@nihon-u.ac.jp (Y.Y.); takahashi.ken08@nihon-u.ac.jp (K.T.); yamaguchi.junko@nihon-u.ac.jp (J.Y.); sakurai.atsushi@nihon-u.ac.jp (A.S.); 2 Department of Pharmacy, Nihon University Itabashi Hospital, Tokyo 173-8610, Japan; imai.toru@nihon-u.ac.jp

**Keywords:** antimicrobial susceptibility, catecholamines, early indicator, norepinephrine equivalent (NEE), septic shock

## Abstract

**Background/Objectives**: The 28-day mortality rate for septic shock is high, necessitating rapid and effective empiric antimicrobial therapy. In this study, we investigate whether the rate of catecholamine dose reduction in septic shock can indicate bacterial susceptibility to initial antimicrobial therapy or not. **Methods:** This retrospective observational study involved 108 adult patients with bacteraemia and septic shock admitted to the intensive care unit of Nihon University Itabashi Hospital between January 2017 and December 2023. They were classified into the Susceptible or Resistant groups based on the bacteria’s susceptibility to the initial empiric antimicrobial therapy. Catecholamine dosages were converted to norepinephrine equivalent (NEE) scores, with the time course from the peak to the end of administration measured at NEE reductions. **Results:** Of the 108 patients, 94 were in the Susceptible group and 14 in the Resistant group. The Susceptible group showed faster reductions in catecholamine doses: the time to reduce the dose from the maximum NEE to 25% was 19 vs. 49.5 h (*p* = 0.0057), and to 0%, it was 29 vs. 54 h (*p* = 0.0475). The time to reduce the dose from the maximum NEE to 75% was 8 vs. 12.5 h (*p* = 0.0733), and to 50% it was 13 vs. 21.5 h (*p* = 0.1081). **Conclusions:** In septic shock with bacteraemia, a faster catecholamine dose reduction indicates bacterial susceptibility to the initial empiric antibiotics. This reduction rate can serve as an early clinical indicator of the appropriate initial empiric therapy.

## 1. Introduction

The 28-day mortality rate for septic shock is as high as 46.5% [1]. Recently, the mortality rate for septic shock in the intensive care unit (ICU), according to the Sepsis-3 definition, is as high as 28.9% [2]. Initial empiric antibiotic therapy is crucial for improving clinical outcomes in septic shock [3]. Some guidelines recommend initiating empiric antibiotic therapy within 1 h [4]. Initiating empiric antibiotic therapy within 3 h has been associated with lower in-hospital mortality rates among patients with sepsis and septic shock [5]. Given the limited time, initial empiric antibiotic therapy for septic shock must be selected based on the causative organism and patient background. Contrarily, inappropriate antimicrobial therapy for sepsis and septic shock is associated with increased mortality [6,7]. Inappropriate initial empiric antibiotic therapy has been observed in 19% of cases of bloodstream infections and is associated with an increased risk of mortality [8]. Although the early determination of the susceptibility of the causative bacteria to the initial antimicrobial agent is desirable, a bacterial culture susceptibility test, which is generally available, requires 2–5 days for results [9]. Even if antimicrobial agents are changed once susceptibility test results reveal antimicrobial resistance, the prognosis remains poor. An early indicator to clinically determine the appropriateness of the initial antimicrobial therapy could facilitate the earlier initiation of effective therapy; however, no such clear early indicator is currently available. We investigated the time course of catecholamine dose reduction in septic shock as a potential early indicator. We hypothesised that antimicrobial susceptibility would result in an early reduction in the catecholamine dose, while antimicrobial resistance would result in a later reduction. We aimed to determine whether the time course of catecholamine dose reduction differs depending on antimicrobial susceptibility or resistance.

## 2. Materials and Methods

### 2.1. Study Design

This retrospective observational study was conducted at the Nihon University Itabashi Hospital, Tokyo. This study included all sequential adult patients admitted to the ICU between 1 January 2017 and 31 December 2023 diagnosed with bacteraemia and septic shock. The Sepsis-3 criteria were used to define septic shock [10]. Bacteraemia was defined as the presence of viable bacteria in blood cultures, after excluding contaminating organisms. Contaminating organisms were determined retrospectively by an infectious disease specialist through a medical chart evaluation. Patients with infections susceptible to initial empiric antimicrobial therapy were classified into the ‘susceptible group’, while those with resistant infections were categorised into the ‘resistant group’. Susceptibility or resistance was determined by the results of the susceptibility test of the bacteria detected in blood cultures. All bacterial cultures and susceptibility testing for this study were conducted in our in-hospital bacteriology laboratory. Matrix-assisted laser desorption ionisation–time-of-flight mass spectrometry (MALDI-TOF MS), a bacterial identification method, has been available in our hospital since 2015. Rapid microbiological susceptibility diagnostics, such as multiplex polymerase chain reaction (PCR) assays, are not used in our hospital.

### 2.2. Exclusion Criteria

The following patients were excluded from this study: those who requested only palliative care, those with cardiopulmonary arrest before ICU arrival, those initially treated at other hospitals or in non-ICU settings, those for whom bacterial susceptibility testing results were not available, and those who died within 24 h of ICU admission. Exclusions due to palliative care were patients who did not wish to receive ICU treatment, artificial organ therapy, or surgery necessary to control infections. Patients who underwent resuscitation for cardiopulmonary arrest prior to ICU admission were excluded because this was an observational study of catecholamine dosing in septic shock. Since the use of catecholamine in cases of cardiopulmonary arrest is related to circulatory failure rather than solely septic shock, excluding these patients was necessary. Patients were excluded if they were admitted to the ICU after catecholamine therapy had already been initiated or they had received ICU or invasive treatment at other hospitals. Patients who were admitted to the ICU after catecholamine therapy had already been initiated and invasive treatment had already been performed in the general wards were also excluded. This was because the time course of catecholamine administration, which was the main objective of this study, was unclear. Patients admitted to the ICU within 6 h of receiving initial treatment alone were not excluded. Patients with unknown susceptibility were excluded. Patients were excluded if susceptibility testing was suspended by the bacteriology laboratory due to early death or if the organism from the blood culture could not be tested for susceptibility. In our hospital, susceptibility testing is discontinued if the patient’s death is confirmed at the time of organism identification. Patients who died within 24 h of ICU admission were excluded, as these early deaths are more likely attributable to the severity of shock, rather than to antimicrobial susceptibility. Furthermore, in our hospital, susceptibility testing is typically discontinued in cases where the patient dies within 24 h of admission.

### 2.3. Norepinephrine Equivalent (NEE)

The norepinephrine equivalent (NEE) formula was used to calculate the total catecholamine dose [11]. The NEE was calculated as follows: norepinephrine (NE) dose (μg/kg/min) + epinephrine (Epi) dose (μg/kg/min) + 0.01 × dopamine (DoA) dose (μg/kg/min) + 0.06 × phenylephrine dose (μg/kg/min) + 2.5 × vasopressin (VA) dose (U/min) + 0.0025 × angiotensin II dose (ng/kg/min) + 10 × terlipressin dose (μg/kg/min) + 0.2 × methylene blue dose (mg/kg/h) + 8 × metaraminol dose (μg/kg/min) + 0.02 × hydroxocobalamin dose (g) + 0.4 × midodrine dose (μg/kg/min). Several studies have utilised NEE formulas [12,13,14], and the NEE formula for 2023 was developed based on these studies. The NEE was calculated for each patient in this study. The maximum (Max) NEE value was defined as Max NEE. The time course from the peak to the end of catecholamine administration was measured using the times at Max NEE; the end of Max NEE; and 75%, 50%, 25%, and 0% of Max NEE. The initial time was defined as the time at which lactate levels > 2 mmol/L were confirmed, and septic shock was diagnosed. The difference between the end of Max NEE time and each subsequent time point was calculated to determine the duration of catecholamine reduction.

### 2.4. Clinical Protocol

In our ICU, blood pressure targets for septic shock are managed above a mean blood pressure (MAP) of 65 mmHg according to international guidelines [15]. As a principle, catecholamines are titrated to maintain an MAP of 65 mmHg–70 mmHg. NE is the first-choice vasopressor: VA is added when MAP cannot be maintained even when the NE dose is higher than 0.1 ug/kg/min. Additionally, low-dose steroids (hydrocortisone 200 mg/day) are administered when the NE dose is higher than 0.1 ug/kg/min to promote early recovery from shock [16].

### 2.5. Sample Collection and Measurement

The following data were collected from clinical records: age, sex, body mass index (BMI), weight, infectious source (urinary tract infection [UTI], pneumonia, soft tissue infection [STI], biliary tract, intra-abdominal, others, and unknown), Gram-stain classification of bacteria (Gram-positive coccus [GPC], Gram-positive rod [GPR], Gram-negative coccus [GNC], and Gram-negative rod [GNR]), ICU outcome (whether the patient survived), sequential organ failure assessment (SOFA) score, blood sampling data, medical history (diabetes and steroid use), and treatment (ventilator use, dialysis, low-dose steroids, and invasive source control). The SOFA score, which measures organ dysfunction, is calculated as a point score based on the function of six organ systems: the respiratory, coagulation, hepatobiliary, cardiovascular, central nervous, and renal systems. A higher score (0–24) is associated with increased ICU mortality [17].

### 2.6. Statistical Analyses

All statistical analyses were conducted using JMP 13.0.0 (SAS Institute Inc., Cary, NC, USA). Normality tests indicated that the data followed a non-normal distribution. The Mann–Whitney U test was used to compare results between the two groups (the Susceptible and Resistant groups). Categorical variables were compared using Fisher’s exact test. Statistical significance was set at *p* < 0.05. The data are presented as the median and interquartile range due to the non-normal distribution. Kaplan–Meier curves were used for the survival analysis of the two groups.

## 3. Results

**Figure 1:**
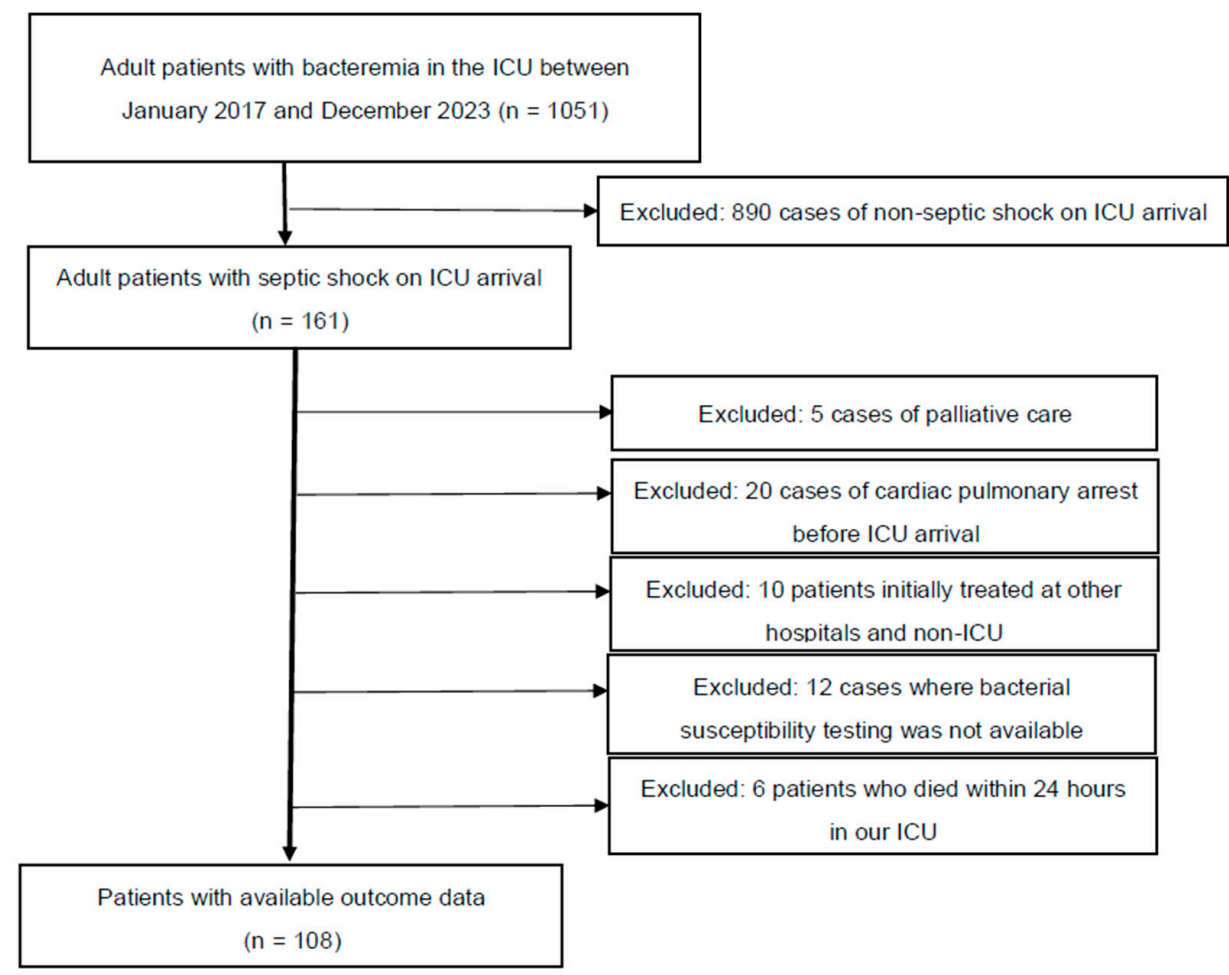
Patient selection flowchart.

Figure 1 presents the patient selection flowchart. A total of 108 patients were sequentially reviewed for analysis. Table 1 presents the clinical characteristics of all 108 patients. Among these patients, 94 and 14 were classified into the Susceptible and Resistant groups, respectively, based on their response to the initial antimicrobial therapy. In terms of ICU survival, 69 (73%) and 8 (57%) patients in the Susceptible and Resistant groups, respectively, survived (*p* = 0.2196). The following characteristics were significantly different between the Susceptible and Resistant groups (interquartile range): ICU stay, 7 (3–13) vs. 3.5 (1–5) days (*p* = 0.0137); median initial lactate level, 5.7 (3.6–7.8) vs. 3.8 (2.0–5.8) mmol/L (*p* = 0.0197); and the coagulation component of the SOFA score, 1 (0–2) vs. 0 (0–1) (*p* = 0.0178). The other characteristics were not significantly different between the two groups. The main treatments for septic shock, including ventilator use, haemodialysis, low-dose steroids, invasive source control, and total infusion volume during the first 24 h, were not significantly different between the two groups. Kaplan–Meier curves based on the Susceptible and Resistant groups are shown in Figure S1 (Wilcoxon, *p* = 0.0818).

All adult patients admitted to the ICU between January 2017 and December 2023 who were diagnosed with bacteraemia and septic shock (*n* = 161) were considered for inclusion. However, 5 patients were excluded due to requests for only palliative care, 20 due to cardiac pulmonary arrest before ICU arrival, 10 for being initially treated at other hospitals and non-ICU settings, and 12 due to the unavailability of bacterial susceptibility testing (in 4 patients, bacterial susceptibility testing was challenging in our bacteriology laboratory, and in 8 cases susceptibility testing was not performed due to early death). Additionally, 4 patients were excluded due to early death within 24 h of ICU admission. Finally, 108 patients were included for analysis (ICU, intensive care unit).

The infection parameters for the Susceptible and Resistant groups are shown in Table 2. The following characteristics were significantly different between the Susceptible and Resistant groups: GPC, 35 (37%) vs. 10 (71%) (*p* = 0.0206); de-escalation, 53 (56%) vs. 0 (0%) (*p* = <0.0001). The 10 patients with GPC in the Resistant group included 6 patients with methicillin-resistant *Staphylococcus aureus* (MRSA), 3 with methicillin-resistant coagulase-negative *Staphylococcus* (MRCNS), and 1 with *Enterococcus faecalis*. The other characteristics were not significantly different between the groups.

**Figure 2:**
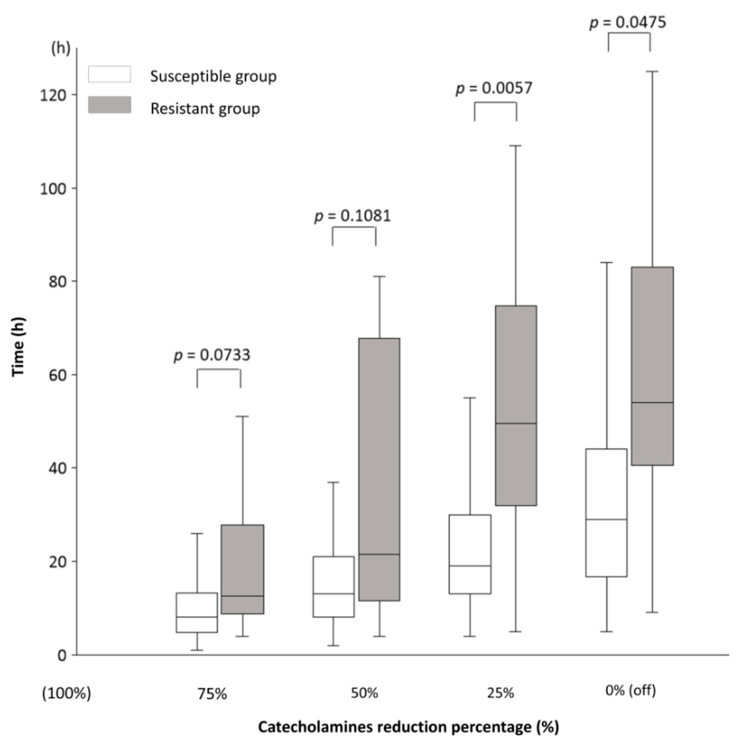
The time course of catecholamine dose reduction in septic shock. The time course from the end of the administration of the maximum (Max = 100%) catecholamine dose to 75%, 50%, 25%, and 0% (off). In the Susceptible and Resistant groups, the time to reduce the dose from 100% to 75% was 8 (5–13) vs. 12.5 (9–28) h (*p* = 0.0733); 50%, 13 (8–21) vs. 21.5 (11.5–68) h (*p* = 0.1081); 25%, 19 (13–30) vs. 49.5 (32–75) h (*p* = 0.0057); and 0% (off), 29 (17–44) vs. 54 (40.5–83) h (*p* = 0.0475). Results are shown as medians and interquartile ranges. The numbers of patients in the Susceptible and Resistant groups for each time point were as follows: 75%, 84 vs. 8; 50%, 83 vs. 8; 25%, 81 vs. 8; and 0%, 80 vs. 7. The decrease in patient numbers was due to deaths during catecholamine administration. There are no missing data. The norepinephrine equivalent (NEE) formula was used to calculate the total catecholamine dose. The NEE was calculated as follows: norepinephrine (NE) dose (μg/kg/min) + epinephrine (Epi) dose (μg/kg/min) + 0.01 × dopamine (DoA) dose (μg/kg/min) + 0.06 × phenylephrine dose (μg/kg/min) + 2.5 × vasopressin (VA) dose (U/min) + 0.0025 × angiotensin II dose (ng/kg/min) + 10 × terlipressin dose (μg/kg/min) + 0.2 × methylene blue dose (mg/kg/h) + 8 × metaraminol dose (μg/kg/min) + 0.02 × hydroxocobalamin dose (g) + 0.4 × midodrine dose (μg/kg/min). Mann–Whitney U test; DoA, dopamine; Epi, epinephrine; NE, norepinephrine; NEE, norepinephrine equivalent; VA, vasopressin.

The main results of this study, which illustrate the time course from the end of Max NEE to 75%, 50%, 25%, and 0% (off) of Max NEE in the Susceptible and Resistant groups, are shown in Figure 2: the time to reach 75% was 8 (5–13) vs. 12.5 (9–28) h (*p* = 0.0733); 50%, 13 (8–21) vs. 21.5 (11.5–68) h (*p* = 0.1081), 25%, 19 (13–30) vs. 49.5 (32–75) h (*p* = 0.0057); and 0%, 29 (17–44) vs. 54 (40.5–83) h (*p* = 0.0475). Table 3 presents the results related to NEE for all participants. The catecholamines used in this study were NE, VA, DoA, and Epi. The amounts of each catecholamine at Max NEE were 0.33 (0.24–0.40) for NE, 0 (0–0.33) for VA, 0 (0–0) for DoA, and 0 (0–0) for Epi. No significant differences in Max NEE and the amounts of each catecholamine were observed between the Susceptible and Resistant groups. The durations from the diagnosis of shock to Max NEE were 9 (3–14) and 11 (6–22) h in the Susceptible (n = 94) and Resistant groups (n = 14), respectively (*p* = 0.1530). The durations from shock diagnosis to the end of Max NEE were 14 (7–22) and 19 (10–48) h in the Susceptible (n = 91) and Resistant groups (n = 10), respectively (*p* = 0.1499). During Max NEE, three patients in the Susceptible group and four in the Resistant group died. In the sensitivity analysis, the same study was conducted excluding patients who died while receiving catecholamines (n = 87). The time course from the end of Max NEE to 75%, 50%, 25%, and 0% (off) of Max NEE in the Susceptible and Resistant groups, excluding deceased patients, is shown in Table 4: the times to reach 25% (19 (13–30) vs. 46 (31–72.5) h, *p* = 0.0152) and 0% (29 (17–44) vs. 54 (40.5–83) h, *p* = 0.0475) were significantly shorter in the Susceptible group than in the Resistant group.

## 4. Discussion

In this study, we demonstrated that, in cases of bacteraemia with septic shock, catecholamine doses were reduced faster in patients with bacteria susceptible to the initial empiric antimicrobial therapy, while the reduction took longer in those with resistant bacteria. To the best of our knowledge, no previous study has examined the rate of catecholamine dose reduction in septic shock, as investigated in the present study.

The reduction to 25% and 0% of the maximum catecholamine dose was significantly faster in the Susceptible group than in the Resistant group. Regarding the rate of catecholamine dose reduction, we consider the time of decrease from the maximum dose to be a clinically understandable indicator in each case, e.g., whether the time of decrease from NE 0.4 μg/kg/min to 0.1 μg/kg/min is within 24 h. The durations from shock diagnosis to Max NEE and the end of Max NEE were not significantly different between the Susceptible and Resistant groups. However, because the duration was slightly longer in the Resistant group, a larger sample size might show a significant difference. If the time to reach peak catecholamine levels is longer in the Resistant group, it may be a potential further early indicator. Further studies with larger sample sizes are needed. The prompt administration of a sensitive antimicrobial agent may lead to a more rapid decrease in catecholamine doses, as it facilitates earlier elimination of causative organisms and quicker infection control in the Susceptible group. In this study, no significant differences were observed in other treatments between the Susceptible and Resistant groups, and the treatment strategies were largely uniform, as they were administered within the same institution. Therefore, it is unlikely that additional factors, such as the impact of steroidal inflammation control, contributed to the reduction in catecholamines. However, the direct reasons for this decrease cannot be definitively determined in this observational study.

Septic shock has a high early mortality rate, and some patients die while on Max catecholamine or during catecholamine dose reduction, which may lead to a survival bias in the analysis of those who survive. To account for potential survival bias, we conducted the same statistical analysis for survivors only as a sensitivity analysis. The results indicate that the Susceptible group experienced a faster decrease in catecholamine doses, regardless of survival status.

In this study, the NEE score represents the total amount of catecholamines administered. The NEE score is a measure of the total amount of catecholamines administered [11]. One study calculating the total amount of catecholamines administered used the same methodology for NE, VA, DoA, and Epi as for the NEE score [14]. Another study applied a different coefficient for DoA (1/150 × DoA dose). However, DoA is not currently recommended for septic shock, and is rarely used [12]. Based on these studies, the NEE score remains a suitable measure for presenting the total amount of catecholamines. In the present study, NE accounted for the largest proportion of NEE, followed by VA. These findings align with current guidelines, which recommend NE and VA as the first and second choices, respectively, of catecholamines for septic shock. Teja et al. also stated that if NE doses approach 0.3 μg/kg/min, VA is initiated for most patients [15,18]. In our ICU, low-dose steroids (hydrocortisone 200 mg/day) are administered when catecholamines are at high doses, and 85% of patients in this study received steroids. Low-dose steroids for septic shock are effective in facilitating early catecholamine reduction [16]. The significantly shorter ICU stay in the Resistant group was due to the high number of early deaths in this group. This is because of antimicrobial resistance to the initial empiric therapy. This finding is consistent with the higher mortality rates observed among patients receiving inappropriate antimicrobial therapy [8]. The significantly lower initial lactate levels in the Resistant group may be attributed to clinicians not using broad-spectrum initial antimicrobial therapy, possibly perceiving the shock to be of milder severity. The significantly higher proportion of GPC among the causative organisms in the Resistant group is thought to be a result of the lack of MRSA and MRCNS coverage in some cases. This suggests that our institution may need to administer more anti-MRSA antibiotics. However, these results cannot be extrapolated to other regions or institutions due to differences in the epidemiology of causative organisms and drug susceptibility, a phenomenon known as the “local factor”. The de-escalation rate was significantly lower in the Resistant group. De-escalation is a change to a narrower spectrum of antimicrobial therapy after antimicrobial susceptibility is confirmed. De-escalation is considered when the antimicrobial susceptibility of the bacteria is known in the Susceptible group. In contrast, de-escalation cannot be performed in the Resistant group at this time. Consequently, the significantly lower de-escalation rate in the Resistant group is a natural outcome. De-escalation therapy is a downstream event that occurs following susceptibility confirmation. Therefore, the De-escalation rate should not be used as an early indicator of antimicrobial efficacy.

If the catecholamine dose reduction rate is slow, such as not reaching 25% of the maximum dose in 24 h, this may indicate antimicrobial resistance. In such cases, clinicians should consider antimicrobial escalation or additional therapies. Appropriate antimicrobial therapy for sepsis improves clinical outcomes [19]. On the other hand, 19% of patients with bacteraemia receive inappropriate treatment, which is associated with higher mortality [8]. Although reducing inappropriate initial empiric antibiotic therapy is essential, selecting the most appropriate antimicrobials within the 1–3 h time frame from the diagnosis of septic shock is challenging. Thus, early clinical indicators of antimicrobial susceptibility are needed. Recent studies have demonstrated that rapid microbiological susceptibility diagnostics, such as multiplex PCR assays, can improve outcomes in bacteraemia. However, the high cost and low versatility of these tests have restricted their availability in many hospitals [20,21]. Sepsis mortality rates vary by region. The highest mortality rates are found in regions that are the least equipped to prevent, identify, or treat sepsis [22]. However, the catecholamine dose reduction rate does not require additional testing and can be determined without an infectious disease specialist. Therefore, the catecholamine dose reduction rate could be a valuable global indicator, regardless of regional disparities. Clinicians should consider antimicrobial escalation if there is only a small reduction in catecholamine dosage, for example, only a 50% or less reduction in 24 h in catecholamine dosage from the end of the catecholamine peak. Depending on future research, an escalation decision may be made earlier than 24 h, and a combined assessment of catecholamine reduction and initial biomarkers may also enhance decision-making. Further research, such as increasing the number of cases or conducting prospective studies, is needed to validate these recommendations.

This study has some limitations. First, the results may not apply to cases of septic shock diagnosed before 2016, since we used data from 2017 to 2023. The Sepsis-3 definition, introduced in 2016, brought some changes to the diagnostic criteria and treatment approaches, such as the early administration of antibiotics, which were not applied before. Second, the sample size, especially in the Resistant group, was very small for the results to be significant, particularly regarding the time to 75% or 50% NEE reduction, where trends were observed but no significant differences emerged. Additionally, due to the limitation of the small sample size, we decided to conduct a simple bivariate analysis instead of a multivariate analysis in this study. Given that this was an exploratory study, future research should focus on increasing the number of patients and conducting multicentre studies to investigate potential institutional biases. Third, this study was conducted in a single-centre ICU, which means that the bacterial strains detected, and their susceptibility may not be generalisable to other facilities. The proportion of the antibiotics used also differs between our facility and others because bacterial susceptibility profiles vary among facilities. However, because the NEE score-based rate of catecholamine dose reduction can be measured easily at any institution, the results of this study may be widely applicable for evaluating antimicrobial susceptibility, irrespective of the specific bacteria or antibiotics used. Fourth, some causative organisms may not have been detected in the blood cultures. Future studies should examine whether similar results are obtained in patients with septic shock other than those with bacteraemia. Fifth, the relationship with biomarkers such as procalcitonin (PCT) and presepsin could not be examined in this study because it was a retrospective study, and the number of biomarker test cases was small. PCT is a biomarker associated with the intensity of the inflammatory response; a meta-analysis of PCT and respiratory tract infections used a PCT-based algorithm to reduce the duration of antimicrobial therapy (8.1 days vs. 9.5 days) without harmful effects [23]. Combining PCT with the speed of NEE reduction in this study may allow for an early determination of antibiotic susceptibility. There are no studies on PCT and the speed of decline of NEE. In future prospective studies, it may be useful to examine the relationship between the speed of catecholamine decrease and the initial biomarker such as PCT, as well as the relationship with antimicrobial susceptibility.

## 5. Conclusions

This study showed that, in cases of bacteraemia with septic shock, catecholamine doses are reduced early when the bacteria are susceptible to initial empiric antimicrobial therapy and later when the bacteria are resistant. Clinicians should consider escalation or additional antibiotics if the catecholamine dose reduction from the maximum dose is slow.

## Figures and Tables

**Table 1:** Clinical characteristics of patients in the Susceptible and Resistant groups.

		Total	Susceptible Group	Resistant Group	*p* Value
	Number	108	94	14	
	Age (years)	78 (70–86)	77 (70–85)	86 (74–90)	0.0608
	Male	64 (59%)	53 (56%)	11 (79%)	0.1498
	BMI, (kg/m^2^)	21 (18–25)	22 (18–25)	20 (17–26)	0.6706
	Weight (kg)	53 (43–65)	54 (44–65)	51 (40–55)	0.2625
Outcome	Survived in ICU	77 (71%)	69 (73%)	8 (57%)	0.2196
	ICU stay (days)	6 (3–11.5)	7 (3–13)	3.5 (1–5)	0.0137
Patient Profile	SOFA score	11.5 (9–13)	12 (9–13)	11 (9–13)	0.5191
	Respiration	2 (1–2)	2 (1–2)	2 (2–3)	0.3060
	Coagulation	1 (0–2)	1 (0–2)	0 (0–1)	0.0178
	Hepatobiliary	0 (0–1)	0 (0–1)	0 (0–1)	0.4969
	Cardiovascular	4 (4–4)	4 (4–4)	4 (4–4)	0.2934
	Central nervous	2 (1–3)	2 (1–3)	2 (1–2)	0.4846
	Renal	2 (1–4)	2 (1–4)	3 (1–4)	0.6333
	Initial lactate (mmol/L)	5.4 (3.4–7.3)	5.7 (3.6–7.8)	3.8 (2.0–5.8)	0.0197
	Initial pH	7.35 (7.28–7.44)	7.35 (7.27–7.44)	7.39 (7.34–7.45)	0.2510
	Initial HCO_3_^−^ (mmol/L)	17.5 (13.1–20.3)	17.0 (12.0–20.1)	19.4 (16.2–23.0)	0.1200
	WBCs (10^4^/µL)	11 (6–18)	11 (6–18)	12 (8–15)	0.7076
	Neutro/Lympho ratio ^1^	17 (9–34)	17 (9–35)	19 (9–21)	0.6657
	CRP (mg/dl)	13 (5–25)	13 (5–26)	9 (4–20)	0.3652
Medical history	Diabetes	47 (44%)	43 (46%)	4 (29%)	0.2621
	Steroid use	11 (10%)	10 (11%)	1 (7%)	1.0000
Treatment	Ventilator use	63 (58%)	56 (60%)	7 (50%)	0.5673
	Haemodialysis	48 (44%)	41 (44%)	7 (50%)	0.7753
	Low-dose steroids	92 (85%)	81 (86%)	11 (79%)	0.4328
	Invasive source control	31 (29%)	27 (29%)	4 (29%)	1.0000
	Total infusion volume in first 24 h, mL	6515 (5325–8412)	6526 (5299–8659)	5831 (5451–7299)	0.5280

^1^ Five patients had missing data. *p* value, Susceptible group vs. Resistant group. The results are expressed as the median (interquartile range) and number (percentages). The SOFA score evaluates the degree of organ dysfunction based on the sum of the total scores for six organ systems: the respiratory, coagulation, hepatobiliary, cardiovascular, central nervous, and renal systems. BMI, body mass index; CRP, C-reactive protein; HCO_3_^−^, bicarbonate; ICU, intensive care unit; neutron/lympho, neutrophils/lymphocytes; SOFA, sequential organ failure assessment; WBCs, white blood cells.

**Table 2:** Infection parameters in the Susceptible and Resistant groups.

		Total	Susceptible Group	Resistant Group	*p* Value
	Number	108	94	14	
Infectious source ^1^	UTI	40 (37%)	36 (38%)	4 (29%)	0.7665
	Pneumonia	18 (17%)	14 (15%)	4 (29%)	0.2651
	STI	16 (15%)	14 (15%)	2 (14%)	0.9524
	Biliary tract	15 (14%)	14 (15%)	1 (7%)	0.4340
	Intra-abdominal	14 (13%)	12 (13%)	2 (14%)	0.8745
	Others ^3^	6 (6%)	4 (4%)	2 (14%)	0.1264
	Unknown	11 (10%)	10 (11%)	1 (7%)	0.6866
Gram-stain classification of causative bacteria ^2^	GPC	45 (42%)	35 (37%)	10 (71%)	0.0206
	GPR	15 (14%)	14 (15%)	1 (7%)	0.6870
	GNC	0	0	0	
	GNR	64 (59%)	59 (63%)	5 (36%)	0.0793
Initial antibiotics ^4^	ABPC/SBT	6	4	2	
	TAZ/PIPC	42	37	5	
	CTX	2	1	1	
	CTRX	18	16	2	
	CFPM	13	11	2	
	MEPM	10	9	1	
	DRPM	9	9	0	
	LVFX	2	1	1	
	TEIC	24	22	2	
	VCM	3	3	0	
	Others	8	7	1	
Time from diagnosis to initial antibiotic administration (min)		75.5 (45–121)	78 (45–125)	62.5 (48–109.5)	0.4643
Additional antibiotics	Cases	26 (24%)	14 (15%)	12 (86%)	0.0555
	Days from initial to additional (day)	2 (2–3)	2 (2–3)	2 (2–3)	0.8656
De-escalation		53 (49%)	53 (56%)	0 (0%)	<0.0001
Total days antibiotics administered (day)		5 (12–16)	12 (7–16)	5 (3–13.5)	0.0654

^1^ Five duplicate cases of the source of infection. ^2^ In 13 patients, multiple bacterial species were detected. ^3^ Others includes two patients with infectious endocarditis, one with intrauterine infection, and one with dialysis shunt infection. ^4^ A total of 79 patients were treated with a single antibiotic, while 67 patients were treated with two or more antibiotics. *p* value, Susceptible group vs. Resistant group. The results are presented as the median (interquartile range) and numbers (percentages). GNC, Gram-negative cocci; GNR, Gram-negative rod; GPC, Gram-positive cocci; GPR, Gram-positive rod; STI, soft tissue infection; UTI, urinary tract infection. ABPC/SBT, ampicillin/sulbactam; TAZ/PIPC, tazobactam/piperacillin; CTX, cefotaxime; CTRX, ceftriaxone; CFPM. cefepime; MEPM, meropenem; DRPM, doripenem; LVFX, levofloxacin; TEIC, teicoplanin; VCM, vancomycin.

**Table 3:** NEE in the Susceptible and Resistant groups.

		Total	Susceptible Group	Resistant Group	*p* Value
	Number	108	94	14	
NEE ^1^	Max NEE	0.39 (0.26–0.46)	0.39 (0.26–0.45)	0.41 (0.27–0.52)	0.4209
	Max NE ^2^	0.33 (0.24–0.40)	0.32 (0.24–0.40)	0.38 (0.24–0.44)	0.2846
	Max VA ^3^	0 (0–0.33)	0 (0–0.33)	0 (0–0.33)	0.7966
	Max DoA ^4^	0 (0–0)	0 (0–0)	0 (0–0)	0.6504
	Max Epi ^5^	0 (0–0)	0 (0–0)	0 (0–0)	0.3793
NEE time (h)	Max from initial	9 (4–16) (n = 108)	9 (3–14) (n = 94)	11 (6–22) (n = 14)	0.1530
	Max last from initial	14 (7–23) (n = 101)	14 (7–22) (n = 91)	19 (10–48) (n = 10)	0.1499

*p* value, Susceptible group vs. Resistant group. The results are presented as the median (interquartile range). ^1^ The NEE was calculated using the following formula: norepinephrine (NE) dose (μg/kg/min) + epinephrine (Epi) dose (μg/kg/min) + 0.01 × dopamine (DoA) dose (μg/kg/min) + 0.06 × phenylephrine dose (μg/kg/min) + 2.5 × vasopressin (VA) dose (U/min) + 0.0025 × angiotensin II dose (ng/kg/min) + 10 × terlipressin dose (μg/kg/min) + 0.2 × methylene blue dose (mg/kg/h) + 8 × metaraminol dose (μg/kg/min) + 0.02 × hydroxocobalamin dose (g) + 0.4 × midodrine dose (μg/kg/min). ^2^ NE = 1 × NE dose (μg/kg/min). ^3^ VA = 2.5 × VA dose (U/min). ^4^ DoA = 0.01 × DoA dose (μg/kg/min). ^5^ Epi = 1 × Epi dose (μg/kg/min). DoA, dopamine; Epi, epinephrine; NE, norepinephrine; NEE, norepinephrine equivalent; VA, vasopressin.

**Table 4:** The time course of catecholamine dose reduction in the Susceptible and Resistant groups, excluding patients who died.

		Total	Susceptible Group	Resistant Group	*p* Value
	Number	87	80	7	
Time to catecholamine reduction from 100% ^1^ (h)					
	75%	9 (5–15)	8 (5–14)	10 (8.5–17.5)	0.2348
	50%	13 (8.5–21)	13 (8–20)	17 (11–51)	0.2541
	25%	20 (13–31.5)	19 (13–30)	46 (31–72.5)	0.0152
	0%	31 (17–47)	29 (17–44)	54 (40.5–83)	0.0475

*p* value, Susceptible group vs. Resistant group. ^1^ The norepinephrine equivalent (NEE) formula was used to calculate the total catecholamine dose. The NEE was calculated as follows: norepinephrine (NE) dose (μg/kg/min) + epinephrine (Epi) dose (μg/kg/min) + 0.01 × dopamine (DoA) dose (μg/kg/min) + 0.06 × phenylephrine dose (μg/kg/min) + 2.5 × vasopressin (VA) dose (U/min) + 0.0025 × angiotensin II dose (ng/kg/min) + 10 × terlipressin dose (μg/kg/min) + 0.2 × methylene blue dose (mg/kg/h) + 8 × metaraminol dose (μg/kg/min) + 0.02 × hydroxocobalamin dose (g) + 0.4 × midodrine dose (μg/kg/min). DoA, dopamine; Epi, epinephrine; NE, norepinephrine; NEE, norepinephrine equivalent; VA, vasopressin.

## Data Availability

The data presented in this study are available on request from the corresponding author due to case privacy.

## Supplementary Materials

The following supporting information can be downloaded at: https://www.mdpi.com/article/10.3390/jcm13216618/s1, Figure S1: Kaplan–Meier curves of the Susceptible and Resistant groups.

## References

[1] Shankar-Hari M., Phillips G.S., Levy M.L., Seymour C.W., Liu V.X., Deutschman C.S., Angus D.C., Rubenfeld G.D., Singer M. (2016). Sepsis Definitions Task Force. Developing a new definition and assessing new clinical criteria for septic shock: For the third international consensus definitions for sepsis and septic shock (Sepsis-3). JAMA.

[2] Shah A.D., MacCallum N.S., Harris S., Brealey D.A., Palmer E., Hetherington J., Shi S., Perez-Suarez D., Ercole A., Watkinson P.J. (2021). Descriptors of sepsis using the Sepsis-3 criteria: A cohort study in critical care units within the U.K. National Institute for Health Research critical care health informatics collaborative. Crit. Care Med..

[3] Im Y., Kang D., Ko R.E., Lee Y.J., Lim S.Y., Park S., Na S.J., Chung C.R., Park M.H., Oh D.K. (2022). Time-to-antibiotics and clinical outcomes in patients with sepsis and septic shock: A prospective nationwide multicenter cohort study. Crit. Care.

[4] Levy M.M., Evans L.E., Rhodes A. (2018). The Surviving Sepsis Campaign Bundle: 2018 update. Intensive Care Med..

[5] Tang F., Yuan H., Li X., Qiao L. (2024). Effect of delayed antibiotic use on mortality outcomes in patients with sepsis or septic shock: A systematic review and meta-analysis. Int. Immunopharmacol..

[6] Vallés J., Rello J., Ochagavía A., Garnacho J., Alcalá M.A. (2003). Community-acquired bloodstream infection in critically ill adult patients: Impact of shock and inappropriate antibiotic therapy on survival. Chest.

[7] Micek S.T., Welch E.C., Khan J., Pervez M., Doherty J.A., Reichley R.M., Kollef M.H. (2010). Empiric combination antibiotic therapy is associated with improved outcome against sepsis due to Gram-negative bacteria: A retrospective analysis. Antimicrob. Agents Chemother..

[8] Kadri S.S., Lai Y.L., Warner S., Strich J.R., Babiker A., Ricotta E.E., Demirkale C.Y., Dekker J.P., Palmore T.N., Rhee C. (2021). Inappropriate empirical antibiotic therapy for bloodstream infections based on discordant in-vitro susceptibilities: A retrospective cohort analysis of prevalence, predictors, and mortality risk in US hospitals. Lancet Infect. Dis..

[9] Miller M.B., Atrzadeh F., Burnham C.A., Cavalieri S., Dunn J., Jones S., Mathews C., McNult P., Meduri J., Newhouse C. (2019). Clinical utility of advanced microbiology testing tools. J. Clin. Microbiol..

[10] Singer M., Deutschman C.S., Seymour C.W., Shankar-Hari M., Annane D., Bauer M., Bellomo R., Bernard G.R., Chiche J.D., Coopersmith C.M. (2016). The third international consensus definitions for sepsis and septic shock (Sepsis-3). JAMA.

[11] Kotani Y., Di Gioia A., Landoni G., Belletti A., Khanna A.K. (2023). An updated “norepinephrine equivalent” score in intensive care as a marker of shock severity. Crit. Care.

[12] Khanna A., English S.W., Wang X.S., Ham K., Tumlin J., Szerlip H., Busse L.W., Altaweel L., Albertson T.E., Mackey C. (2017). Angiotensin II for the treatment of vasodilatory shock. N. Engl. J. Med..

[13] Laterre P.F., Berry S.M., Blemings A., Carlsen J.E., François B., Graves T., Jacobsen K., Lewis R.J., Opal S.M., Perner A. (2019). Effect of selepressin vs placebo on ventilator- and vasopressor-free days in patients with septic shock: The SEPSIS-ACT randomized clinical trial. JAMA.

[14] Goradia S., Sardaneh A.A., Narayan S.W., Penm J., Patanwala A.E. (2021). Vasopressor dose equivalence: A scoping review and suggested formula. J. Crit. Care.

[15] Evans L., Rhodes A., Alhazzani W., Antonelli M., Coopersmith C.M., French C., Machado F.R., Mcintyre L., Ostermann M., Prescott H.C. (2021). Surviving sepsis campaign: International guidelines for management of sepsis and septic shock 2021. Intensive Care Med..

[16] Rygård S.L., Butler E., Granholm A., Møller M.H., Cohen J., Finfer S., Perner A., Myburgh J., Venkatesh B., Delaney A. (2018). Low-dose corticosteroids for adult patients with septic shock: A systematic review with meta-analysis and trial sequential analysis. Intensive Care Med..

[17] Vincent J.L., de Mendonça A., Cantraine F., Moreno R., Takala J., Suter P.M., Sprung C.L., Colardyn F., Blecher S. (1998). Use of the SOFA score to assess the incidence of organ dysfunction/failure in intensive care units: Results of a multicenter, prospective study. Working group on “sepsis-related problems” of the European Society of Intensive Care Medicine. Crit. Care Med..

[18] Teja B., Bosch N.A., Walkey A.J. (2023). How we escalate vasopressor and corticosteroid therapy in patients with septic shock. Chest.

[19] Ferrer R., Martin-Loeches I., Phillips G., Osborn T.M., Townsend S., Dellinger R.P., Artigas A., Schorr C., Levy M.M. (2014). Empiric antibiotic treatment reduces mortality in severe sepsis and septic shock from the first hour: Results from a guideline-based performance improvement program. Crit. Care Med..

[20] Perez K.K., Olsen R.J., Musick W.L., Cernoch P.L., Davis J.R., Peterson L.E., Musser J.M. (2014). Integrating rapid diagnostics and antimicrobial stewardship improves outcomes in patients with antibiotic-resistant Gram-negative bacteremia. J. Infect..

[21] Okamoto M., Maejima M., Goto T., Mikawa T., Hosaka K., Nagakubo Y., Hirotsu Y., Amemiya K., Sueki H., Omata M. (2023). Impact of the FilmArray rapid multiplex PCR assay on clinical outcomes of patients with bacteremia. Diagnostics.

[22] Rudd K.E., Johnson S.C., Agesa K.M., Shackelford K.A., Tsoi D., Kievlan D.R., Colombara D.V., Ikuta K.S., Kissoon N., Finfer S. (2020). Global, regional, and national sepsis incidence and mortality, 1990–2017: Analysis for the Global Burden of Disease Study. Lancet.

[23] Schuetz P., Wirz Y., Sager R., Christ-Crain M., Stolz D., Tamm M., Bouadma L., Luyt C.E., Wolff M., Chastre J. (2017). Procalcitonin to initiate or discontinue antibiotics in acute respiratory tract infections. Cochrane Database Syst. Rev..

