# Supplementary material for: The Time Course of Catecholamine Dose Reduction in Septic Shock as a Predictor of Bacterial Susceptibility to Empiric Antimicrobial Therapy: A Retrospective Observational Study
Source: J Clin Med. 2024 Nov 4;13(21):6618. doi: 10.3390/jcm13216618 (PMC11546866; doi:10.3390/jcm13216618)
Supplement: Supplementary file 1 [file jcm-13-06618-s001.zip › jcm-3208034-supplementary.pdf]

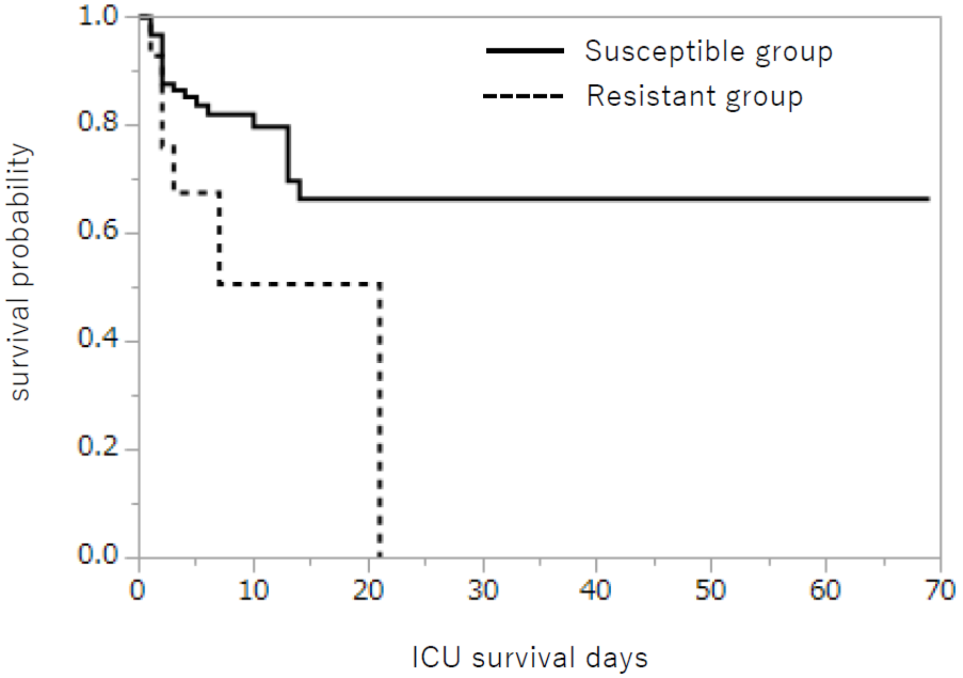

Figure S1. Kaplan-Meier curves of the Susceptible and Resistant group. (Wilcoxon, p = 0.0818). ICU, intensive care unit.
